# Sustainable approach for catalytic green epoxidation of oleic acid with applied ion exchange resin

**DOI:** 10.1038/s41598-023-42879-4

**Published:** 2023-09-19

**Authors:** Mariam Abdul Rahman, Nabisab Mujawar Mubarak, Intan Suhada Azmi, Mohd Jumain Jalil

**Affiliations:** 1 Chemical Engineering Studies, College of Engineering, Universiti Teknologi MARA Cawangan Pulau Pinang, Kampus Permatang Pauh, Perai, Malaysia; 2 Petroleum and Chemical Engineering, Faculty of Engineering, Universiti Teknologi Brunei, Bandar Seri Begawan, BE1410 Brunei Darussalam; 3 Department of Biosciences, Saveetha School of Engineering, Saveetha Institute of Medical and Technical Sciences, Chennai, India; 4 Chemical Engineering Studies, College of Engineering, Universiti Teknologi MARA Cawangan Johor, Kampus Pasir Gudang, Masai, Malaysia

**Keywords:** Environmental sciences, Environmental social sciences, Chemistry, Energy science and technology

## Abstract

Epoxides were primarily derived from petroleum-based sources. However, there has been limited research on optimizing the process parameters for epoxidized palm oil-derived oleic acid, resulting in its underutilization. Therefore, this study aimed to optimize the catalytic epoxidation of palm oleic acid concerning the oxirane content by applying ion exchange resin as a catalyst. Epoxidized oleic acid was produced using in-situ-formed performic acid by combining formic acid as the oxygen carrier with hydrogen peroxide as the oxygen donor. The findings revealed that the optimal reaction conditions for producing epoxidized oleic acid with the highest oxirane content were an Amberlite IR-120 catalyst loading of 0.9 g, a molar ratio of formic acid to oleic acid of 1:1., and a molar ratio of hydrogen peroxide to oleic acid of 1:1.1. By employing these optimal conditions, the maximum relative conversion of palm oleic acid to oxirane was achieved at 85%. The reaction rate constants (k) based on the optimized epoxidized oleic acid are determined as follows: k_11_ = 20 mol L^−1^ min^−1^, k_12_ = 2 mol L^−1^ min^−1^, and k_2_ = 20 mol L^−1^ min^−1^. The findings validated the kinetic model by showing good agreement between the simulation and experimental data.

## Introduction

Palm oil is commonly used in the modern era due to its many benefits and advantages as a renewable resource rather than petroleum, which will also decrease in general^1^. Palm oil has increased demand for various products and has become one of the largest exportations in the world. Epoxidation of palm oil is known as the carbon atoms adjacent to the alkyl group that is joined to the same oxygen atom in the structure, and these products are named epoxides or oxiranes^2^. In the epoxidation process, two methods are known in industrial peracids: peracetic acid^3^ and performic acid^4^. At present, the most common feedstock used is derived from petroleum. However, petroleum-derived feedstocks are costly and detrimental to the environment. In the past few years, the utilization of epoxidized vegetable oils has become more common because epoxides can act as chemical precursors for the synthesis of numerous chemicals such as alcohols, glycols, alkanol amines, carbonyl compounds, olefinic compounds, and polymers such as polyesters, polyurethanes, and epoxy resins^5–7^. Producing epoxidized oil from renewable resources has become important due to concerns about sustainability and global warming.

Although several parts of the epoxidation of vegetable oil problem have been identified, there is a lack of studies showing how the epoxidation process occurs in palm oil via ion exchange resin as a catalyst (Amberlite IR-120). In the previous study, much research was conducted on using acid catalysts such as sulfuric acid for the epoxidation process. It can lead to corrosion since sulfuric acid is a strong acid in an oxidizing environment^8^. Besides, acid catalysts can also cause decreased oxirane stability which is important since the epoxidation reaction is very unstable, and the active intermediate products can easily lead to ring opening^9^. Enzymes are biological catalysts that can facilitate various chemical reactions, including epoxidation. Enzyme-catalyzed epoxidation reactions are often more environmentally friendly and selective than traditional chemical methods^10^. It is also crucial to investigate the effect of each process parameter on the overall epoxidation process.

Amberlite IR-120 are insoluble gel catalysts in the form of small yellowish organic polymer beads. Peroxy acid is obtained from a reaction between hydrogen peroxide and carboxylic acid. The peroxy acid interacts with the catalyst by seeping through the pores of the catalyst^11^. Thus, when the amberlite IR-120 is loaded into the reactor, the pores of amberlite IR-120 are filled with peracid. This reduces oxirane degradation because the triglycerides enter the gel structure of the amberlite IR-120 catalyst^12^. Many studies were carried out to investigate the conversion of unsaturated fatty acids into oxirane rings using peracid (either performic acid or peracetic acid) with the presence of an amberlite IR-120 catalyst^13^. The results show that different vegetable oils have different degrees of conversion. Goud et al.^14^ performed in situ epoxidation of Karanja oil with aqueous peroxide and acetic acid in the presence of an amberlite IR-120 catalyst. The parameters studied were the catalyst loading, hydrogen peroxide, temperature, molar ratio of double bonds to oxirane groups, and reaction time. The optimum molar ratio of acetic acid to Karanja oil was 0.5, and the maximum for hydrogen peroxide was 1.5.

Therefore, the objectives of this study were to investigate the effect of catalyst concentration, hydrogen peroxide to oleic acid molar ratio, and formic acid to oleic acid molar ratio on the relative conversion rate to oxirane. The developed kinetic model of epoxidation of palm oleic acid with applied amberlite IR-120 as a catalyst to determine the reaction rate.

## Materials and method

### Material

The Merck Sdn. Bhd. has provided the aqueous formic acid with 85% concentration, hydrogen peroxide with 30% and 50% concentration, and sulphuric acid catalyst with 95% purity. Additionally, crystal violet, hydrogen bromide with 48% concentration, and glacial acetic acid with 100% purity were titrated. The crude oleic acid contains 75% oleic acid, 12% linoleic acid, 6% palmitic acid and 6% stearic acid. All the composition was tested by gas chromatography technique.

### Experimental Set-up- Epoxidation of oleic acid

The experiment was conducted in a 500-mL beaker utilizing a hot plate as the heat source. Initially, 100 g of oleic acid was mixed with formic acid in a specific molar ratio (1.0, 1.5, or 2.0) and added to the beaker equipped with a magnetic stirrer and thermometer. The beaker was then placed in a thermostatic water bath, and the mixture was stirred using an overhead stirrer at 300 rpm and heated to a temperature of 70 °C. Hydrogen peroxide was added drop by drop to the mixture in an amount corresponding to a specific molar ratio of hydrogen peroxide to oleic acid (1.0, 1.5, or 2.0). Subsequently, 0.2 g of Amberlite IR-120 catalyst was added to the mixture, which was then allowed to react for 1 h. Finally, a 4 g sample was withdrawn using a syringe for oxirane content analysis, following the American Oil Chemists Society (AOCS) Official Method Cd-957.

#### Reusability test for Amberlite IR-120 catalyst

The reusability of a catalyst refers to its ability to be used multiple times in a chemical reaction without significant loss of its catalytic activity. A reusability test is conducted in the second cycle of Amberlite IR-120 as a catalyst by filtering it from the mixture, cleaning using acetone or hexane, and heating it in the oven to the desired temperature (130 °C) for 1 h. Then, the epoxidation was carried out again to measure the level of reduction in relative conversion to oxirane (RCO). The accepted reusable catalyst only when the difference RCO for the first and second limit to less than 10%. The reusability of the catalyst can be evaluated by comparing the activity of the catalyst in subsequent reactions to its activity in the initial reaction. The reusability studies identify the process's economic feasibility, and the reaction's environmental impact can be minimized.

#### Determination relative conversion to oxirane (RCO)

RCO is a term used to describe the extent of epoxidation in a reaction. RCO is the ratio of epoxide produced in the reaction to the initial amount of double bonds in the reactant^15^. Wet analysis is a type of analysis that involves the measurement of the amount of a substance in a sample by direct or indirect weight determination. In the case of RCO determination, wet analysis can involve measuring the amount of epoxide in a sample by weighing the sample before and after the reaction or by using a chemical reaction to convert the epoxide to a known compound and then measuring the amount of the known compound produced. Wet analysis is a common method used for RCO determination in epoxidation reactions, as it directly and accurately measures the amount of epoxide produced^16^. The results of the wet analysis can be used to calculate the RCO, which can then be used to evaluate the effectiveness of the epoxidation reaction and to optimize the reaction conditions for improved performance.

The experiment estimated the RCO by theoretically calculating the oxirane oxygen content (OOC) and empirically calculating OOC using a hydrobromic (HBr) acid solution for titration. The RCO Eq. (1) includes both the theoretical (2) and empirical (3) OOC values.

1 $$RCO = \frac{{OOC_{expiment} }}{{OOC_{theoretical} }} \times 100$$

2 $$OOC_{the} = \left\{ {{{\left( {\frac{{X_{0} }}{{A_{i} }}} \right)} \mathord{\left/ {\vphantom {{\left( {\frac{{X_{0} }}{{A_{i} }}} \right)} {\left[ {100 + \left( {\frac{{X_{0} }}{{2A_{i} }}} \right)\left( {A_{o} } \right)} \right]}}} \right. \kern-0pt} {\left[ {100 + \left( {\frac{{X_{0} }}{{2A_{i} }}} \right)\left( {A_{o} } \right)} \right]}}} \right\} \times A_{o} \times 100$$

3 $$OOC_{Exp} = 1.6\; \times \;N\; \times \;\frac{{\left( {V - B} \right)}}{W}$$

where X_O_ is the initial iodine value, A_i_ is the molar mass of iodine, A_O_ is the molar mass of oxygen, N is the normality of HBr, V is the volume of HBr solution used for the titration, and W is the weight of the sample.

#### Identification of best process parameters of epoxide production

Identifying the best process parameters for producing epoxidized oleic acid is essential for maximizing yield and quality, reducing costs and waste, increasing efficiency, and ensuring consistency in the production process. Table 1 shows the production of epoxidized oleic acid.

**Table 1:** Parameters involved in the production of epoxidized oleic acid.

Materials	Range/amount	Purity/molarity
Oleic acid	100 g	75%
The formic acid molar ratio	0.5, 1.0, 1.5	23.6 M
Catalyst Concentration	0.3,0.6,0.9	Wt%
Hydrogen peroxide molar	0.5, 1.0, 1.5	50%

#### Fourier transform infrared (FTIR) spectroscopy

FTIR spectroscopy is a powerful analytical technique for identifying and analyzing materials' molecular structure and chemical composition^17^. In FTIR spectroscopy, a beam of infrared light is passed through a sample, which absorbs specific wavelengths of the light depending on the functional groups and chemical bonds present in the sample. The resulting infrared spectrum is then used to identify and analyze the sample. In this study, the functional groups of the samples were identified using an FTIR spectrometer (Spectrum One, PerkinElmer, USA). The crude oleic acid and acid were analyzed using FTIR spectroscopy to determine the presence of epoxide groups and the disappearance of absorption peaks (ascribed to =CH stretching). The FTIR spectra were recorded within a wavenumber range of 400–4000 cm^−1^, the mid-infrared region corresponding to the fundamental vibration modes of the molecules.

#### Numerical kinetic model of epoxidation of oleic acid

A kinetic study is involved in the degradation of the epoxidation of palm oil. Moreover, the information based on the kinetic study can be simulated using MATLAB software to determine the value of the kinetic rate equation, which involves the derivatives or differentials of the dependent variable. The differentials are presented as ordinary differential equations. MATLAB is one of the standard solvers used to solve ordinary differential equations (ODEs). The epoxidation process is characterized by two main reactions (the in situ formation of performic acid and epoxidized oleic acid, as indicated by Eqs. (4) and (5), respectively).

4 $$FA + HP \begin{array}{*{20}c} {\to ^{k1} } \\ {\mathop \leftarrow \limits_{k2} } \\ \end{array} PFA + H_{2} O$$

5 $$PFA + OA \to ^{k3} Epoxide + FA$$

FA, HP, and PFA are formic acid, hydrogen peroxide and performic acid, respectively.

6 $$\frac{{d\left[ {FA} \right]}}{dt} = - k_{11} \left[ {FA} \right]\left[ {HP} \right] + k_{12} \left[ {PFA} \right]\left[ {Water} \right] + k_{2} \left[ {PFA} \right]\left[ {OA} \right]$$

7 $$\frac{{d\left[ {HP} \right]}}{dt} = - k_{11} \left[ {FA} \right]\left[ {HP} \right] + k_{12} \left[ {PFA} \right]\left[ {Water} \right]$$

8 $$\frac{{d\left[ {PA} \right]}}{ dt} = + k_{11} \left[ {FA} \right]\left[ {HP} \right] - k_{12} \left[ {PFA} \right]\left[ {Water} \right] - k_{2} \left[ {PFA} \right]\left[ {OA} \right]$$

9 $$\frac{{d\left[ {Water} \right]}}{dt} = + k_{11} \left[ {FA} \right]\left[ {HP} \right] - k_{12} \left[ {PFA} \right]\left[ {Water} \right]$$

10 $$\frac{{d\left[ {OA} \right]}}{dt} = - k_{2} \left[ {PFA} \right]\left[ {OA} \right]$$

11 $$\frac{{d\left[ {Epoxide} \right]}}{dt} = + k_{2} \left[ {PFA} \right]\left[ {OA} \right]$$

To determine the rate coefficient numerically, parametric studies were performed. This requires two computational procedures: the numerical solution of a set of differential equations (Eqs. 6–11) and the calculation of the errors between experiment and simulation. The error function e is given by Eq. (12).

12 $$e = \mathop \sum \limits_{i = 1}^{n} \frac{{\left| {EPOA_{i}^{sim} - EPOA_{i}^{exp} } \right|}}{n}$$

## Result and discussion

### Effect of amberlite IR-120 concentration as a catalyst on epoxidation

**Figure 1:**
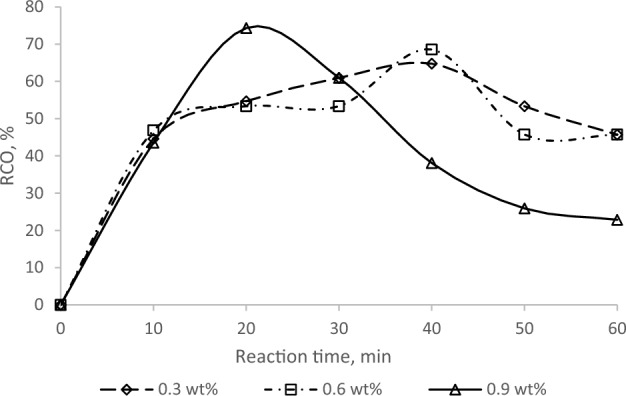
Effect of concentration amberlite IR-120H on epoxidation.

This study aims to investigate the effectiveness of using an ion exchange resin catalyst (amberlite IR-120) as a safer and more environmentally friendly alternative to traditional homogenous (sulfuric acid) and heterogenous (zeolite, titanium) catalysts in the epoxidation of palm oleic acid to produce epoxidized oleic acid. Results from Fig. 1 show that the highest RCO value of 77.5% at 20 min was achieved with an Amberlite concentration of 0.9 wt%, which resulted in less opening of the epoxidized oleic acid ring compared to 0.3 wt% and 0.6 wt%. The effect of catalyst loading on the RCO was also explored, with an increase in catalyst loading generally leading to an increase in reaction rate and RCO. However, too high a catalyst concentration can lead to side reactions, by-product formation, and decreased product quality due to catalyst particle agglomeration blocking active sites^18^. The catalyst accelerates the process and reduces energy consumption and operational costs^19^.

The active sites the catalyst provides facilitate the formation of performic acid from formic acid and hydrogen peroxide, which then reacts to epoxidized oleic acid. Using 0.9% Amberlite IR-120 resulted in a high RCO due to the acid’s hygroscopic nature. Catalyst and palm oleic acid serve as substrates for further epoxidation, as the generation of performic acid is a reversible process. However, suppose the equilibrium balance favors performic acid creation is not achieved. In that case, performic acid breaks down, leading to a decrease in its concentration and an increase in the water content of the reaction system^20^. Thus, the catalyst is crucial in achieving the highest rate and conversion. Therefore, a catalyst loading of 0.9% was used in the subsequent experiment to obtain the highest product yield. Vegetable oils have been subjected to epoxidation using acidic catalysts such as sulfuric acid and hydrochloric acid. The main function of these catalysts is to protonate the carbon–carbon double bond present in the unsaturated fatty acid, thus making it more vulnerable to attack by a peroxide^21^.

### Effect of formic acid/oleic acid molar ratio on epoxidation

**Figure 2:**
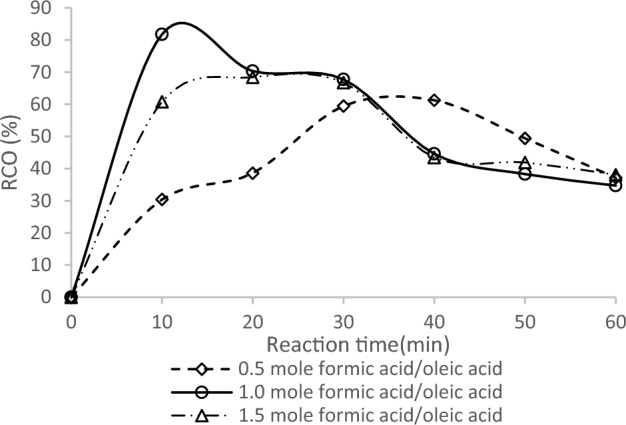
Effect of formic acid molar ratio on epoxidation.

The impact of the formic acid molar ratio on the rate of oleic acid epoxidation is depicted in Fig. 2 for 0.5 and 1.0-mol formic acid, respectively, with respect to the RCO. The highest RCO of 81.5% was attained in 10 min of reaction time for the formic acid molar ratio of 1.0, while the lowest RCO of 62% was observed in 31 min for the formic acid molar ratio of 0.5. The study examined three formic acid/oleic acid molar ratios (0.5, 1.0, and 1.5), and the impact of this factor on the RCO is displayed in Fig. 2. pH was evaluated based on the formic acid/oleic acid molar ratios, as formic acid supplies hydrogen ions. Initially, the RCO values were nearly the same for both molar ratios. However, after approximately 15 min, the RCO values began to differ. Nonetheless, the maximum RCO obtained was comparable for both molar ratios, with a value of 81% (molar ratio 1.0) and 62% (molar ratio 0.5).

The experiment was conducted at a reaction temperature of 70 °C and a stirring speed of 300 rpm. Epoxidized vegetable oils act as chemical processors during oxirane ring degradation, forming more by-products and openings^22^. The RCO was lower for a molar ratio of 0.5, possibly due to degradation occurring at a higher rate. Avoiding a high formic acid concentration is important since it can lead to oxirane ring degradation, which is unstable in an acidic environment. As evidenced by the decrease in RCO values after a reaction time of 45 min, an increase in formic acid concentration promotes oxirane ring opening by hydrolysis.

### Effect of hydrogen peroxide molar ratio on epoxidation

**Figure 3:**
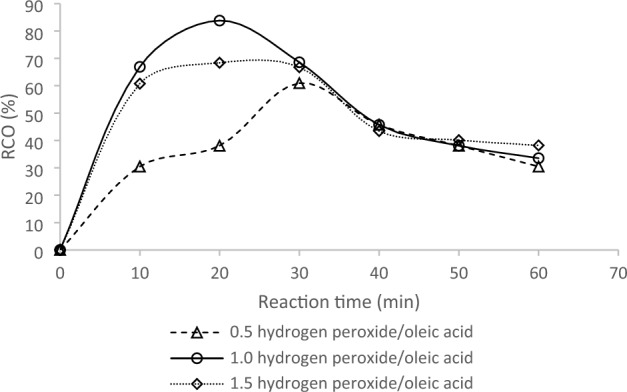
Effect of hydrogen peroxide molar ratio on RCO.

**Figure 4:**
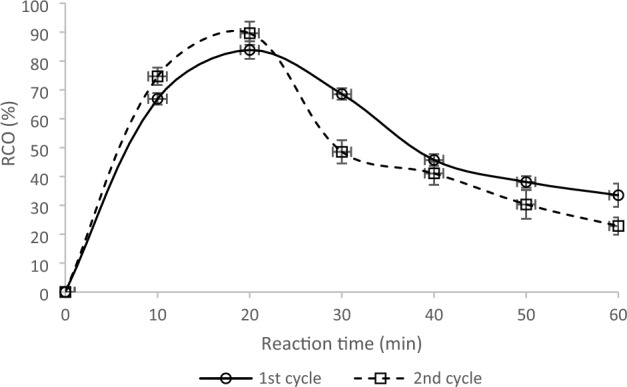
Reusability test for catalyst.

Figure 3 illustrates the impact of different hydrogen peroxide molar ratios on the RCO, with the Amberlite IR 120 concentration fixed at 0.9 wt%. The hydrogen peroxide molar ratios investigated in this study were 0.5, 1.0, and 1.5. The results revealed that the highest RCO of 85% was achieved after a reaction time of 20 min when the hydrogen peroxide molar ratio was 1.0. The RCO values obtained were 68% and 61% when the hydrogen peroxide molar ratios were 1.5 and 0.5, respectively, and the reaction times were 30 and 40 min. The use of various mole ratios of hydrogen peroxide to oleic acid (1:1, 1:1.5, and 1:1.64) for the formation of epoxidized oleic acid was investigated, as shown in Fig. 4. The hydrogen peroxide acts as an oxygen donor during the formation of the oxirane ring^23^. The results indicated that increasing the hydrogen peroxide concentration resulted in a higher percentage of RCO. However, the lowest mole ratio of hydrogen peroxide to oleic acid (1:1) exhibited poor stability for the oxirane ring. It is worth noting that a low hydrogen peroxide concentration may increase the degradation rate, resulting in the formation of diol and α-glycol as side products, as reported in a previous study^24^.

A previous study suggested that a mole ratio of 1:0.75 of aqueous hydrogen peroxide to oleic acid gives the highest epoxidation rate, with a 30% concentration^25^. However, this concentration of hydrogen peroxide is susceptible to instability, resulting in high rates of ring degradation and the production of diol and α-glycol as side products. In conclusion, although a high concentration of hydrogen peroxide can increase the rate of oxirane conversion, it can also negatively affect the stability of the oxirane.

### Reusability test of catalyst

The reusability test of a catalyst refers to evaluating its ability to maintain its catalytic activity after repeated use in a chemical reaction. The goal is to determine if the catalyst can still efficiently and effectively facilitate the reaction after multiple cycles without significant performance loss. The test typically involves measuring the catalyst’s activity, stability, and selectivity before and after each cycle of use. As shown in Fig. 4, the results of the first and second cycles are comparable, and there are no significant differences; it could be a positive outcome regarding cost-effectiveness and sustainability.

Amberlite is a brand of ion exchange resins commonly used as a catalyst in the epoxidation reaction. In this reaction, an alkene molecule is converted into an epoxide by adding an oxygen atom. The Amberlite catalyst acts as a Lewis acid, promoting the formation of a reaction intermediate that leads to the epoxide product. Amberlite ion exchange resins are known for their high stability, selectivity, and reusability, making them a popular choice for industrial epoxidation processes. The exact type of Amberlite catalyst used for a particular epoxidation reaction depends on the requirements, such as reaction conditions and the alkene substrate. The reusability of the Amberlite catalyst can be tested by measuring its activity and selectivity after multiple cycles of use in the reaction.

### Brunauer–Emmett–Teller (BET)-catalyst characterization

Nitrogen adsorption studies were conducted to determine the Brunauer–Emmett–Teller (BETsurface area (ABET) and pore volume (Vp) of both catalysts. Table 2 provides a summary of the ABET and Vp amberlite IR-120. As shown in Table 1, amberlite IR-120 exhibited a larger BET surface area and pore volume. This characteristic suggests that the amberlite IR-120 catalyst has more active sites for the epoxidation process, resulting in higher reaction conversion. From the analysis of the amberlite IR-120, the surface area and pore volume were 88 m^2^ g^−1^ and mm^3^ g^−1^, respectively.

**Table 2:** Estimated reaction rates parameters from the simulation.

The rate constant of the epoxidation process	The value rate constant (mol L^−1^ min^−1^)
k_11_	20
k_12_	2
k_2_	20
The correlation coefficient, R_2_	
0.85	

### FTIR spectroscopy

**Figure 5:**
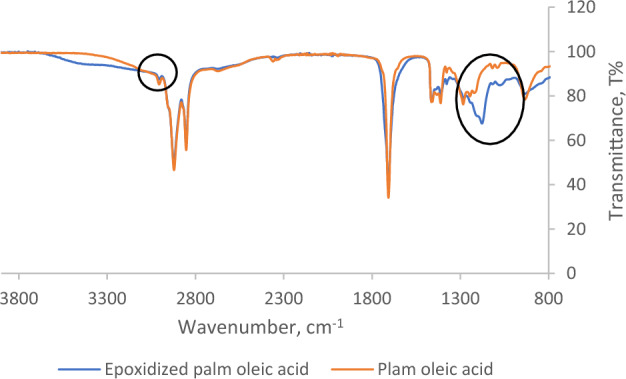
FTIR Spectrum for oleic acid and epoxidized oleic acid.

The chemical properties of crude oleic acid and epoxidized palm oleic acid were examined using FTIR spectroscopy. The FTIR spectra of oleic acid and epoxidized palm oleic acid are presented in Fig. 5. Generally, an oxirane ring can be detected in the 750–880 cm^−1^ wavenumber range. Moreover, the =CH stretching vibration and HC=CH bonding peaks are usually observed in 3050–3000 cm^−1^ and 1700–1600 cm^−1^, respectively. In the FTIR spectra of epoxidized palm oleic acid, the unsaturated =CH stretching vibration peak identified in crude oleic acid was not observed, and the presence of an oxirane ring was confirmed at 1200 cm^−1^.

### Reaction rate determination

Kinetic modelling was performed using MATLAB software to determine the optimal reaction conditions for the epoxidation process, and the reaction rate values, $k$, are summarized in Table 2. The reaction rates, $k$, of the experimental data correspond to the initial concentration for all chemicals. Here, $k$ denotes a specific reaction rate. The higher the value, the faster the reaction. It can be seen from Table 2 that the coefficient. ${k}_{11}$ is significantly higher compared with ${k}_{12}$. Therefore, it is easier to control the loss of performic acid upon adding oleic acid into the in situ-formed performic acid to form epoxides. This speed up the reaction as less energy is required for the molecules to react. Table 2 shows the reaction rate from the simulation.

**Figure 6:**
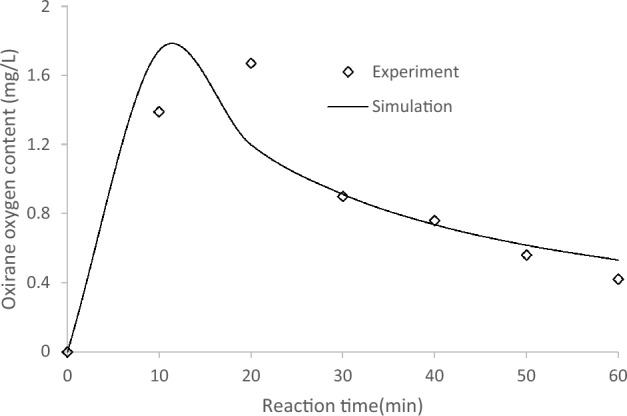
Comparison of oxirane oxygen content from experiment and simulation.

Comparing the experimental and simulated data, there is generally good agreement with a difference of approximately 0.20, as shown in Fig. 6. However, the simulated results show a noticeable deviation between the two datasets for the epoxide formation within the 10 to 20-min range. It’s important to note that an assumption is made when comparing simulation and experimental data. Unlike real-world experiments, the numerical simulation represents an idealized behavior as it does not consider heat loss or heat transfer during the reaction. The simulation relies solely on the chemical equation, assuming all reactions occur simultaneously. This difference arises due to the distinct nature of simulations and experiments.

## Conclusion

In conclusion, energy is crucial in achieving stable socio-economic development, as it influences the price of goods and services. This study investigated the effect of the molar ratio of hydrogen peroxide and formic acid to palm oleic acid and catalyst loading on the epoxidation of palm oleic acid formed in situ. The reaction was carried out using titration at a moderate agitation speed of 350 rpm and a temperature of 80 °C. The optimal relative conversion to oxirane yield of sunflower-based polyol was 85%, achieved through a 1:1:1 formic acid and hydrogen peroxide to palm oleic acid mole ratio reaction with 0.9% Amberlite IR-120 as a catalyst. The main contributions of this study are the significant improvement in the epoxidation process, where the conversion of palm oleic acid from a low-value product into a value-added product—lastly, the development of an eco-friendlier with high efficiency of epoxidized oleic acid production method utilizing ion exchange resin method as the catalyst.

## Data Availability

The datasets used and analyzed during the current study are available from the corresponding author upon reasonable request.
